# Functionalized MoS_2_ Nanoflowers with Excellent Near-Infrared Photothermal Activities for Scavenging of Antibiotic Resistant Bacteria

**DOI:** 10.3390/nano11112829

**Published:** 2021-10-25

**Authors:** Lulu Liu, Wanfeng Wu, Yan Fang, Haoqiang Liu, Fei Chen, Minwei Zhang, Yanan Qin

**Affiliations:** 1 College of Life Science & Technology, Xinjiang University, Urumqi 830046, China; 202112544@mail.sdu.edu.cn (L.L.); wwfeng1207@sina.com (W.W.); fangyan1227@sina.com (Y.F.); 17699101675lhq@sina.com (H.L.); chen970701@sina.com (F.C.); 2 Xinjiang Key Laboratory of Biological Resources and Genetic Engineering, Urumqi 830046, China

**Keywords:** antibiotics resistant bacteria, MoS_2_ nanoflowers, antibacterial

## Abstract

Presently, antibiotic resistant bacteria (ARB) have been commonly found in environment, such as air, soil and lakes. Therefore, it is urgent and necessary to prepare antimicrobial agents with excellent anti-antibiotic resistant bacteria. In our research, poly-ethylene glycol functionalized molybdenum disulfide nanoflowers (PEG-MoS_2_ NFs) were synthesized via a one-step hydrothermal method. As-prepared PEG-MoS_2_ NFs displayed excellent photothermal conversion efficiency (30.6%) and photothermal stability. Under 808 nm NIR laser irradiation for 10 min, the inhibition rate of tetracycline-resistant *Bacillus tropicalis* and *Stenotrophomonas malphilia* reached more than 95% at the concentration of 50 μg/mL. More interestingly, the photothermal effect of PEG-MoS_2_ NFs could accelerate the oxidation of glutathione, resulting in the rapid death of bacteria. A functionalized PEG-MoS_2_ NFs photothermal anti-antibiotic resistant system was constructed successfully.

## 1. Introduction

In recent years, the increase of antibiotic resistant bacteria (ARB) had a great impact on public health [1,2,3,4]. ARB can be continuously spread through the medium of animal excreta, water, and air. In particular, more than a hundred kinds of resistance genes can be detected in the samples collected from livestock and poultry breeding sites. The antibiotic resistant genes of these antibiotic resistant bacteria may be transferred from environmental host bacteria to pathogens through gene horizontal transfer, or from pathogens to primary host bacteria in the environment [5,6,7,8]. It is urgent and necessary to explore and develop non-antibiotic antibacterial agents.

Currently, nanomaterials have received a lot of attention because of their excellent and unique physicochemical properties [9]. Compared with antibiotics, nanomaterials can inhibit ARB through intertwining and membrane perturbation [10], “sharp” edges [11], oxidative stress [12], photothermal ablation etc. [13]. The special antibacterial mechanism enables nanomaterials to avoid the emergence of ARB and lay a certain foundation for their use in inhibiting antibiotic resistant bacteria [14,15]. Carbon-based nanomaterials [12], silver nanoparticles [16,17,18], titanium dioxide nanomaterials [19] and so on have been widely researched for antibacterial. For example, Wahab et al. demonstrated that a nanostructure by incorporating Ag NPs with nanoporous carbon nitride possessed antibacterial effect for both wild type and the multidrug-resistant *Escherichia coli* [17]. Among many nanomaterials, two-dimensional nanomaterials are widely used in antibacterial due to their unique electronic, physical, and chemical properties [20]. In particular, molybdenum disulfide nanomaterials have a very broad prospect of biological application, due to the low toxicity, high near-infrared (NIR) absorbance [21,22], excellent photothermal conversion efficiency [23], and peroxidase-like activity [13,24]. MoS_2_ is connected by molybdenum atoms and sulfur atoms in a covalent bond to form an S-Mo-S sandwich structure, and the layers are stacked together by a weak van der Waals force [25], the S-Mo configuration possesses excellent adsorption [26]. Chou et al. reported for the first time that MoS_2_ has an excellent near-infrared photothermal effect due to the exceptional surface-area-to-mass ratio can load with high cargo concentrations. Interestingly, its extinction coefficient is 7.8 times that of graphene oxide [27]. It is worth knowing that the photothermal effect of flower-like MoS_2_ nanoflakes could be efficiently destroyed cancer cell [28]. In addition, Yang et al. found that MoS_2_ has good antibacterial properties due to membrane stress and reactive oxygen species (ROS) pathways [29]. Membrane composed of chitosan and MoS_2_ also exhibit good antibacterial capability and outstanding antifouling property [30].

**Figure 1:**
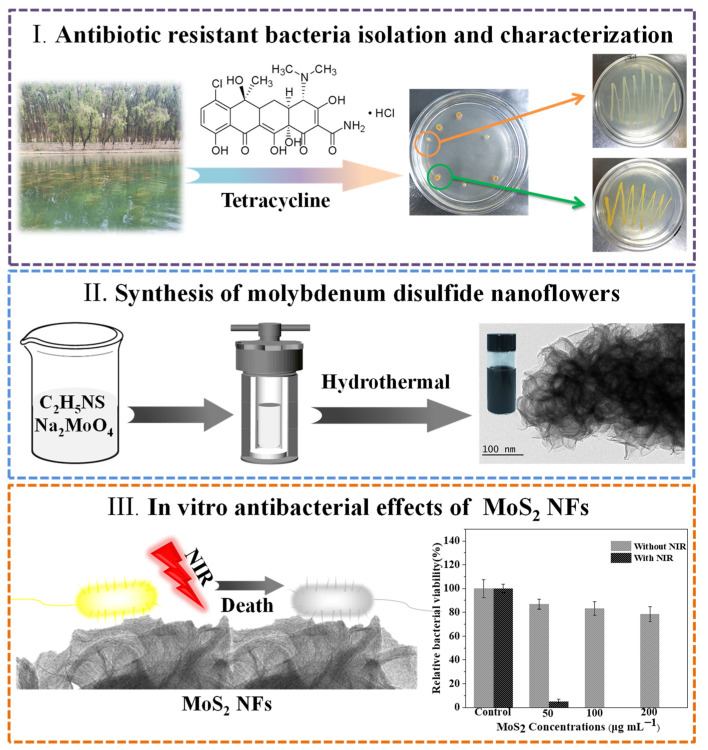
Schematic of PEG-MoS_2_ NFs was constructed for scavenging of ARB.

In this study, polyethylene glycol functionalized molybdenum disulfide nanoflowers (PEG-MoS_2_ NFs) were constructed via a one-step hydrothermal method for scavenging of ARB. First, tetracycline-resistant bacteria were isolated and identified from Honghu Lake water which in Xinjiang University (42°45′32″ N, 86°37′33″ E) and selecting *Stenotrophomonas maltophilia* (*S. maltophilia*) and *Bacillus tropicalis* (*B. tropicalis*) for subsequent antibacterial experiments. Afterwards, the one-step hydrothermal method was used to synthesize PEG-MoS_2_ NFs and the photothermal stability and photothermal conversion efficiency of it were tested. Finally, the excellent photothermal performance of PEG-MoS_2_ NFs under 808 nm near-infrared irradiation was used to study the inhibition of ARB, as shown in Figure 1.

## 2. Materials and Methods

### 2.1. Materials

Yeast powder, peptone, TRIS hydrochloride, and agar powder were purchased from Beijing Soleibao Technology Co., Ltd. Polyethylene glycol (*M*_W_ = 200), sodium molybdate dihydrate and thioacetamide were purchased from Shanghai Aladdin Industrial Company. The bacterial genomic DNA extraction kit was purchased from Tiangen Biochemical Technology Co., Ltd. (Beijing, China). Glutathione was purchased from Shanghai Yuanye Biotechnology Co. Ltd. Sodium chloride was purchased from Tianjin Guangfu Technology Development Co., Ltd. All solvents and reagents were of analytical grade and were used without further purification. Distilled water was used in all experiments. In addition, the apparatuses are given in Supplementary Materials.

### 2.2. Antibiotic Resistant Bacteria Isolation and Characterization

The water samples (collected through the five-point sampling method from Honghu lake Urumqi, Xinjiang, 42°45′32″ N, 86°37′33″ E) were thoroughly suspended in 9 mL sterile physiological saline (0.9%) by vortex, and then 100 μL of the 10-fold serial dilution sample was coated onto solid Luria−Bertani (LB) agar plate including 5 μg/mL tetracycline (TCr, purchased from Soleibao Technology Co., Beijing, China). The single colony was cultured eventually in 5 mL of liquid LB culture medium to isolate tetracycline resistance bacteria in the TCr at 37 °C, 200 rpm overnight and determine the minimum inhibitory concentration (MIC) of TCr for tetracycline resistance bacteria. Consistent with the manufacturerʹs instructions, Bacterial genome DNA was extracted with Bacterial Genome DNA Extraction Kit (TIANGEN, Beijing, China).

The 16s rRNA gene primers used were 5′-AGAGTTTGATCCTGGCTCAG-3′ (Forward primer) and 5′-GGTTACCTTGTTACGACTT-3′ (Reverse primer). The quality of 16s rRNA gene was determined by 1.5% agarose gel electrophoresis. Subsequently, the 16s rRNA gene was sequenced by Bioengineering (Shanghai) Co and analyzed with BLAST tool. In addition, the phylogenetic tree of tetracycline resistance bacteria was generated by MEGA7.0.

### 2.3. Synthesis of Molybdenum Disulfide Nanoflowers

PEG-MoS_2_ NFs were synthesized according to previous reports with a slight modification [16]. Briefly, 0.12 g of thioacetamide and 0.06 g of sodium molybdate dihydrate were added to 40 mL of 50% (*v*/*v*) polyethylene glycol 200 solution (diluted with ultrapure water), and the solution was transferred to a polyphenylene-lined stainless steel autoclave (180 °C, 24 h). After the reaction, the PEG-MoS_2_ NFs products were washed with ultrapure water, vacuum freeze drying and stored at room temperature.

### 2.4. Photothermal Effect of PEG-MoS_2_ NFs

#### 2.4.1. Effect of Concentration on Photothermal Effect of PEG-MoS_2_ NFs

The PEG-MoS_2_ NFs dispersions with the concentrations of 30 μg/mL, 50 μg/mL, 100 μg/mL, 150 μg/mL, and 200 μg/mL were prepared, and 1 mL of the dispersion was transferred to a 2 mL quartz cuvette, and the PEG-MoS_2_ NFs dispersions with different concentrations were irradiated vertically with an 808 nm NIR exciter for 16 min. The temperature change of the dispersions under radiation was recorded every 10 s using a digital thermometer at the same time. Pure water was used as the control group.

#### 2.4.2. Effect of Power on Photothermal Effect of PEG-MoS_2_ NFs

PEG-MoS_2_ NFs dispersions with a concentration of 100 μg/mL were prepared, 1 mL of the dispersions were transferred to a 2 mL quartz cuvette, and the PEG-MoS_2_ NFs dispersions were irradiated vertically with different power densities of an 808 nm NIR exciter for 16 min at a working distance of 9 cm. Meanwhile, the temperature change of the aqueous dispersion under radiation was recorded every 10 s using a digital thermometer.

#### 2.4.3. Photothermal Stability Test

The PEG-MoS_2_ NFs dispersion was prepared at a concentration of 400 μg/mL, and 1 mL of PEG-MoS_2_ NFs dispersion was transferred to a 2 mL quartz cuvette and irradiated vertically with NIR light at a wavelength of 808 nm and a power density of 1.5 W/cm^2^ for 16 min, and then the light source was turned off and cooled down for 16 min. After 16 min, the photothermal warming was then continued. A digital thermometer was used to record the temperature changes during the eight cycles of temperature rise and fall, and the temperature was recorded every 10 s. Finally, the temperature diagram was plotted for photothermal stability analysis.

#### 2.4.4. Photothermal Conversion Efficiency

The photothermal conversion efficiency was calculated by the following equation [22,31].

$$\eta \;=\frac{\;\mathrm{hS}\left(T_{\max}-T_{\mathrm{surr}}\right)-\mathrm{Qdis}}{I(1-10^{A_{\lambda }})}$$

h stands for the heat transfer coefficient, S stands for the surface area of the container, T_max_ and T_surr_ respectively represent the equilibrium temperature and the temperature of the surrounding environment, Q_dis_ are the heat-related to light absorption, I is the incident laser power, and A_λ_ is the absorbance of PEG-MoS_2_ NFs at 808 nm.

### 2.5. In Vitro Antibacterial Effects of PEG-MoS_2_ NFs

#### 2.5.1. Preparation of Bacterial Suspensions

The strains frozen in the −40 °C refrigerator were thawed in an ice water bath and inoculated into liquid LB culture medium after placing the culture tubes in a constant temperature shaking incubator (37 °C, 150 rpm) to activate the expanded bacteria. Then, 500 μL of the activated bacterial suspensions was aspirated into a centrifuge tube containing 5 mL of liquid LB culture medium and incubated in a constant temperature shaking incubator at 37 °C for 5–6 h. After that, the concentration of the bacterial suspensions was adjusted with PBS solution, mixed well and then measured the absorbance value at 600 nm with UV spectrophotometer to make the OD600 of 0.05 and then diluted to 10^−3^ and set aside.

#### 2.5.2. In Vitro Antibacterial Effect of NIR Photothermal Therapy

The antibacterial activity of PEG-MoS_2_ NFs at different concentrations against drug-resistant bacteria was measured by plate counting method. The bacterial suspension (100 μL) obtained from 2.5.1 was mixed with as-prepared nanoparticles with the final concentrations of PEG-MoS_2_ NFs in the reaction system were 0 μg/mL, 50 μg/mL, 100 μg/mL and 200 μg/mL, respectively. The system was mixed thoroughly with 0.01 mol/L PBS solution to 1 mL, and two parallel systems were set up for each group. Group A was placed in a thermostatic shaking incubator for 30 min at 37 °C and 40 μL was aspirated onto solid LB medium culture; Group B was treated with an 808 nm NIR exciter at an operating current of 1.88 A, a power density of 1 W/cm^2^, and an operating distance of 9 cm for 10 min, and then placed in a thermostatic shaking incubator for 20 min at 37 °C and 40 μL was aspirated onto solid LB medium culture.

### 2.6. Ellman’s Assay

Ellman’s assay was used to examine Glutathione (GSH) oxidation [29,32]. Ellman reagent (5,5′-dithiobis (2-nitrobenzoic acid)) will react with thiol groups (–SH) in GSH obtaining a yellow product (2-nitro-5-thiobenzoate acid) by cracking its disulfide bonds (–S–S–). Different concentrations of PEG-MoS_2_ NFs (30, 50, and 100 μg/mL) and 0.8 mM GSH are mixed in a 1.5 mL centrifuge tube in equal volume ratio, and the solvent is bicarbonate buffer solution (50 mM, pH = 8.7), 37 °C, 150 rpm react for 2, 4, and 6 h under dark conditions. 1 mmol/L H_2_O_2_ + GSH was positive control, and GSH was negative control. Afterward, 785 μL of Tris-HCl (0.05 M, pH = 8) solution and 15 μL DTNB (100 mM) was added into the mixture and the PEG-MoS_2_ NFs were removed by centrifuging at 12,000 rpm for 10 min. Finally, the absorbance of the filtrate was measured at 410 nm. To explore the depletion of GSH at temperatures similar to the photothermal treatment, the concentration of PEG-MoS_2_ NFs was set at 100 μg/mL, and the reactions were carried out at 37 °C and 50 °C in the dark in a water bath for 5 min, 10 min, 20 min, 30 min, 60 min, and 90 min, respectively, and the other steps were the same as above.

## 3. Results and Discussion

### 3.1. Identification of Antibiotic Resistant Bacteria

**Figure 2:**
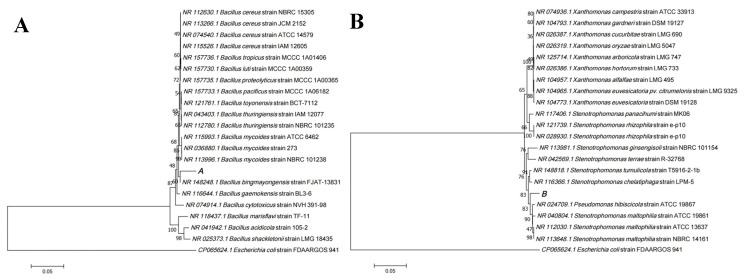
Neighbor-joining tree of strains based on 16s rRNA sequences, showing the respective homology of strain (**A**) and strain (**B**).

The 16s rRNA sequences of the screened ARB were compared with the sequences in the GenBank database using the BLAST tool in NCBI, and the genera of the corresponding strains were identified based on the similarity of the comparison. In addition, Gram-positive (Figure S1A) antibiotic resistant B. tropicusand and the more common clinical Gram-negative (Figure S1B) conditional pathogens *S. maltophilia* were selected from them as the targets for subsequent in vitro inhibition experiments. The phylogenetic tree of the two strains (Labeled as A and B, respectively) was constructed using the neighbor-joining method in MEGA 7.0 software, as shown in Figure 2. Among them, strain A was 97.62% similar to B. tropicus strain MCCC 1A01406 and strain B was 98.67% similar to *S. maltophilia* strain ATCC 13637. In addition, according to the physiological and biochemical tests, both strains were found to be acid-producing and non-gas-producing bacteria (Figure S2). Meanwhile, the minimum inhibitory concentration (MIC) of two strains to tetracycline was 75 μg/mL. This indirectly indicates that the water in Xinjiang University’s Honghu Lake (42°45′32″ N, 86°37′33″ E) is more seriously affected by antibiotics.

### 3.2. Synthesis and Characterization of Molybdenum Disulfide Nanoflowers

**Figure 3:**
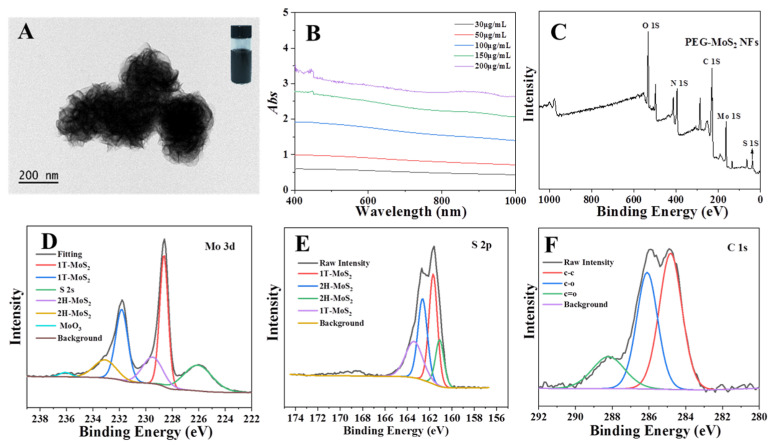
(**A**) TEM images of the PEG-MoS_2_ NFs. (**B**) The absorption spectra of the PEG-MoS_2_ NFs from visible light to the NIR region. (**C**) XPS spectra of PEG-MoS_2_ NFs. (**D**) XPS spectra of Mo 3d orbits. (**E**) XPS spectra of S 2p orbits. (**F**) XPS spectra of C 1s orbits.

PEG-MoS_2_ NFs with good dispersion in water (Top right inset in Figure 3A) were synthesized by a one-step hydrothermal method. Transmission electron microscopy (TEM, JEM-2100, JEOL Co., Tokyo, Japan) (Figure 3A) reveals that the PEG-MoS_2_ NFs exhibits flower-like morphology by stacking MoS_2_ nanosheets, which is consistent with previous reports [28]. As can be seen in Figure 2B, the solution of PEG-MoS_2_ NFs possessed a strong absorption from visible light to the NIR region. The absorbance value showed a clear linear relationship with the concentrations of PEG-MoS_2_ NFs (Figure S3), when the concentration changed from 30 μg/mL to 200 μg/mL, its absorbance value at 808 nm increased from 0.48 to 2.75. The atomic ratio of C:S:Mo in the PEG-MoS_2_ NFs is about 3:2:1 as determined by X-ray photoelectron spectroscopy (XPS, ESCALAB250Xi, Thermo, Waltham, MA, USA) (Figure 3C), while an amorphous layer of carbon consisting of C-C, C-O, and C=O bonds were present on the surface of the PEG-MoS_2_ NFs (Figure 3F). These functional groups producing from the thermal oxidation of PEG adsorbed on the surface of nanoflower by physically or chemically action [33]. In addition, the presence of amorphous carbon could lead to a better dispersion in aqueous. As shown in Figure 3D–E, the XPS peak regions of Mo 3d and S 2p were deconvolved for PEG-MoS_2_ NFs, respectively. The peaks at 228.4 eV and 231.6 eV for the spin-orbit coupling binding energy belong to 3d_5/2_ and 3d_3/2_ of 1T- MoS_2_, respectively, and the peaks at 229.5 eV and 233.2 eV belong to 2H-MoS_2_ (Figure 3D). Moreover, the peaks at 161.1 and 162.6 eV (Figure 3E) originate from S 2p_3/2_ and S 2p_1/2_ of the 2H MoS_2_, respectively. In addition, the peaks at 161.7 eV and 163.4 eV belong to 1T-MoS_2_. 1T-2H mixed MoS_2_ will behave the excellent photothermal performance due to the introducing of 1T-MoS_2_ [34]. All evidence indicated that well dispersed MoS_2_ nanoflowers were successful prepared via one-step hydrothermal.

**Figure 4:**
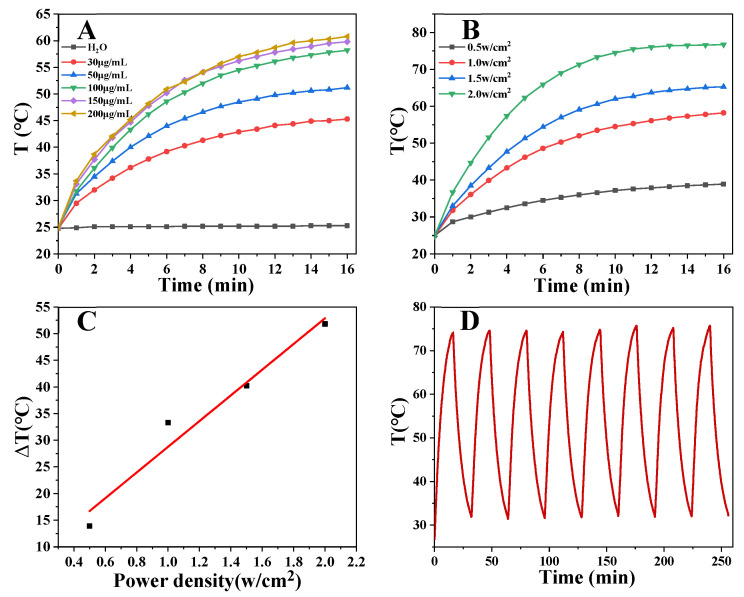
(**A**) Photothermal temperature profiles of PEG-MoS_2_ NFs dispersions and pure water at different concentrations (30 μg/mL, 50 μg/mL, 100 μg/mL, 150 μg/mL, 200 μg/mL) irradiated at power density of 1 W/cm^2^ for 16 min in the NIR; (**B**) The concentration of 100 μg/mL of PEG-MoS_2_ NFs dispersion irradiated at different power densities (0.5 W/cm^2^, 1.0 W/cm^2^, 1.5 W/cm^2^, 2.0 W/cm^2^) for 16 min; (**C**) Linear fit of temperature change (ΔT) to irradiation power density; (**D**) Temperature profiles of PEG-MoS_2_ NFs dispersion with a concentration of 400 μg/mL for continuous switching of the NIR exciter monitoring curve.

**Figure 5:**
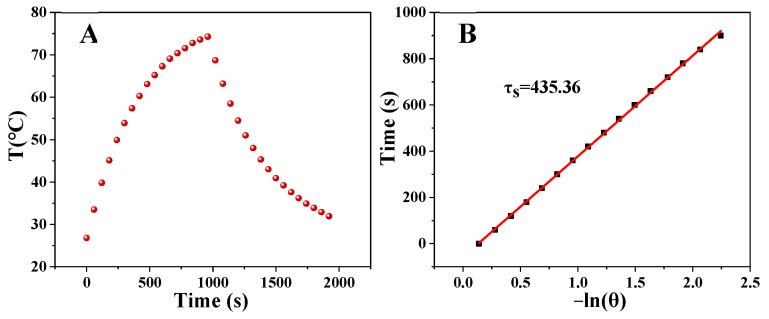
(**A**) Photothermal effect of PEG-MoS_2_ NFs before and after irradiation for 16 min by 808 nm laser at a power density of 1.5 W/cm^2^. (**B**) Graph of the relationship between the cooling time (after 16 min) and the negative natural logarithm of the driving force temperature obtained from the cooling phase.

PEG-MoS_2_ NFs can be used as a photothermal agent to convert the light energy of NIR into heat energy and generate local high temperature. Therefore, the photothermal performance of PEG-MoS_2_ NFs was tested. Various concentrations of PEG-MoS_2_ NFs and different laser power densities were studied when irradiated with 808 nm NIR laser for 16 min. It is noteworthy to mention that the photothermal performance of PEG-MoS_2_ NFs had obvious concentration and power density dependence (Figure 4A–C). The temperature gradually increased with the increase of concentration and power density. When the concentration was 200 µg/mL and the power density was 2.0 W/cm^2^, the temperature was more than 70 °C. PEG-MoS_2_ NFs with a concentration of 400 µg/mL was subjected to a cyclic temperature rise and fall test under a power density of 1.5 W/cm^2^ NIR irradiation to observe the photothermal stability of PEG-MoS_2_ NFs. As shown in Figure 4D, after the eight cycles, the temperature of PEG-MoS_2_ NFs could reach the initial temperature value, proving that the PEG-MoS_2_ NFs material exhibit eminent photothermal stability. Meantime, based on Figure 5A,B, the photothermal conversion efficiency of PEG-MoS_2_ NFs was calculated to be 30.6%, which is higher than that of many other photothermal nanomaterials, as shown in Table 1. Showing PEG-MoS_2_ NFs have excellent photothermal conversion performance.

### 3.3. In Vitro Antibacterial Effects of PEG-MoS_2_ NFs

**Figure 6:**
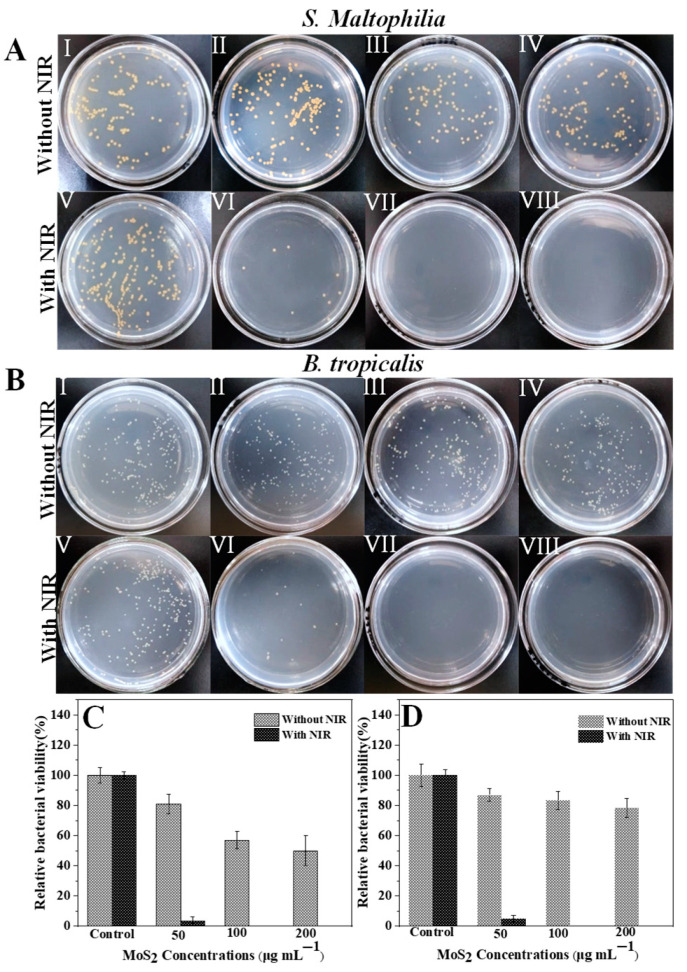
Photographs of bacterial colonies formed by (**A**) *S. Maltophilia* and (**B**) *B. tropicalis* after exposed to (I) PBS, (II) 50 μg/mL PEG-MoS_2_ NFs, (III) 100 μg/mL PEG-MoS_2_ NFs, (IV) 200 μg/mL PEG-MoS_2_ NFs, (V) PBS + NIR, (VI) 50 μg/mL PEG-MoS_2_ NFs + NIR, (VII) 100 μg/mL PEG-MoS_2_ NFs + NIR, (VIII) 200 μg/mL PEG-MoS_2_ NFs + NIR; The relative viability of (**C**) *S. Maltophilia* and (**D**) *B. tropicalis* after heat treatment with 808 nm NIR light for 10 min was measured by plate counting (Error bars are standard deviations of three parallel experiments).

As-prepared PEG-MoS_2_ NFs displayed excellent photothreamal properties; it is an ideal agent for scavenging of ARB. Therefore, the inhibitory effects of PEG-MoS_2_ NFs on antibiotic resistant *S. maltophilia* and antibiotic resistant *B. tropicalis* were evaluated by the plate counting method when irradiated with 1 W/cm^2^ 808 nm NIR laser for 10 min (Figure 6A−D). The results demonstrated that separate PEG-MoS_2_ had no distinct effect on bacterial viability. However, it is of particular interest to obvious that PEG-MoS_2_ could more efficiently decrease the bacterial survival rate and the inhibition rate on both *S. maltophilia* and *B. tropicalis* could reach 100% when adopting the photothermal performance of PEG-MoS_2_. In contrast, there only slight bacteria damage when bacteria with NIR only. Han et al. found that the survival rate of *E. coli* will further decrease with the NIR irradiation of Graphene oxide nanocomposite hydrogels [41]. The results indicated that the PEG-MoS_2_ NFs characteristic with comparable inhibition ability on Gram-positive and Gram-negative antibiotic resistant bacteria mainly due to its excellent photothermal performance.

### 3.4. Oxidation and Quantification of GSH

To further investigate its antibacterial mechanism, the oxidation of glutathione was studied. Glutathione is composed of glycine, cysteine, and glutamic acid, which has an antioxidant effect, and as an antioxidant, it can effectively resist the oxidative damage caused by the oxidative stress from outside to the substances inside the cell. The sulfhydryl group on cysteine is its active group, and when the sulfhydryl group is exposed to reactive oxygen or other oxidants, it can be oxidized into disulfide bonds in a short time, so that the proteins and enzymes are destroyed and the internal disorder of the cell leads to cell death [42]. Therefore, the degree of glutathione oxidation in vitro is an indirect indicator of whether an antimicrobial agent will have an oxidative damaging effect on antibiotic resistant bacteria. In this paper, an Ellman’s assay was used to determine the possibility of PEG-MoS_2_ NFs mediated non-dependent oxidative stress of reactive oxygen species (ROS).

**Figure 7:**
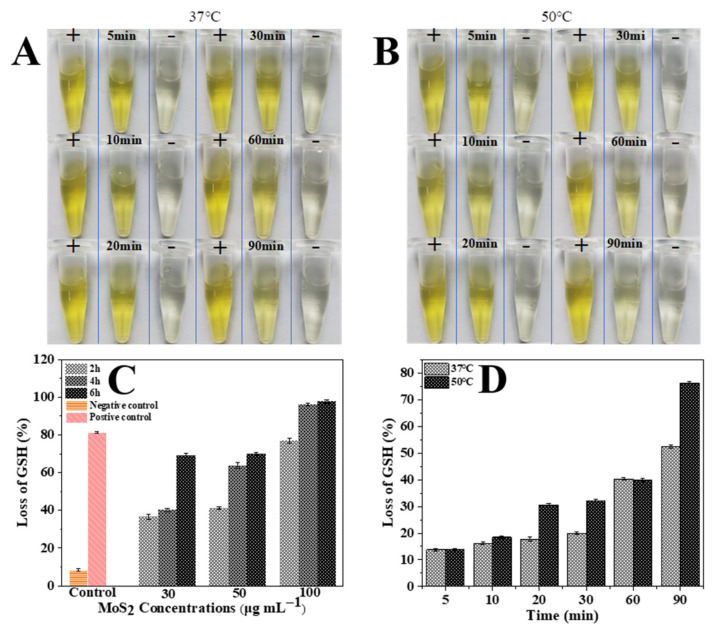
Effect of PEG-MoS_2_ NFs on glutathione oxidation at different reaction times (5 min, 10 min, 20 min, 30 min, 60 min, 90 min) at 37 °C (**A**) and 50 °C (**B**), respectively. (**C**) GSH loss rates of PEG-MoS_2_ NFs dispersions with different concentrations at 37 °C for different reaction times. (**D**) GSH loss rates of PEG-MoS_2_ NFs dispersions with a concentration of 100 μg/mL at 37 °C and 50 °C for different reaction times, respectively.

Typically, PEG-MoS_2_ NFs showed a significant time-dependent oxidation behavior, the loss rate of GSH increased with reaction time (Figure 7C,D). Meanwhile, temperature-dependent GSH oxidations were further implemented to study the influence of heat. The loss rate reached 52.53 ± 0.60% and 76.3 ± 0.51% when the PEG-MoS_2_ NFs dispersions with a concentration of 100 μg/mL were reacted at 37 °C and 50 °C, respectively, which showed the loss rate of GSH increased with higher temperature (Figure 7D). When the 808 nm NIR was irradiated vertically with a power density of 1 W/cm^2^ at a concentration of 100 μg/mL PEG-MoS_2_ NFs dispersion for 16 min, the temperature could reach 58.2 °C, which was higher than those corresponding data obtained at 50 °C. This indicated that PEG-MoS_2_ NFs can enhance the oxidation of GSH and cause rapid death of ARB due to the disorder inside the bacterial cells.

## 4. Conclusions

In conclusion, PEG-MoS_2_ NFs, a bacterial inhibitor with excellent photothermal conversion properties was synthesized by one-step hydrothermal method. The PEG-MoS_2_ NFs has excellent photothermal conversion performance and photothermal stability. Then the inhibitory effect of PEG-MoS_2_ NFs was investigated against *B. tropicalis* and *S. maltophilia* screened from the lake water. The results showed that PEG-MoS_2_ NFs had comparable inhibitory effects on Gram-positive antibiotic resistant and Gram-negative antibiotic resistant bacteria, and the inhibition rate reached 100% when the concentration of PEG-MoS_2_ NFs was 100 μg/mL. Ellman’s assay was carried out to further explained the reason for rapid bacterial death: in the presence of PEG-MoS_2_ NFs, the high temperature-induced by 808 nm NIR laser had a strong promotion effect on the oxidation of GSH, which could cause rapid death of antibiotic resistant bacteria by disrupting the intercellular protection system. This work shows that PEG-MoS_2_ NFs have excellent photothermal antibacterial activity, which provides a fast and effective method for the removal of antibiotic resistant bacteria from the environment.

## Figures and Tables

**Table 1:** The photothermal conversion efficiency of different photothermal nanomaterials.

Photothermal Nanomaterials	Wavelength	Photothermal Conversion Efficiency	Ref.
MoS_2_ nanosheets	808 nm	24.37%	[21]
Silicon phthalocyanine -graphene oxide	808 nm	20.20%	[35]
Rose-bengal-conjugated gold nanorods	810 nm	21%	[36]
Mo_2_C nanospheres	1064 nm	24.95%	[37]
Cu_9_S_5_ nanocrystals	980 nm	25.7%	[38]
Ligand-stabilized copper selenide nanocrystals	800 nm	22%	[39]
copper selenide nanoparticles	980 nm	18.9%	[40]
PEG-MoS_2_ NFs	808 nm	30.6%	This work

## Data Availability

Not applicable.

## Supplementary Materials

The following are available online at https://www.mdpi.com/article/10.3390/nano11112829/s1, Figure S1: The gram staining results of *Bacillus tropicus* (A) and *Stenotrophomonas maltophilia* (B). Figure S2: The acid and gas production test of *Bacillus tropicus* (B) and *Stenotrophomonas maltophilia* (C). Figure S3 Linear relationship between the concentration of PEG-MoS2 NFs dispersion and absorbance values at 808 nm.
