# Correction: Liu et al. Functionalized MoS_2_ Nanoflowers with Excellent Near-Infrared Photothermal Activities for Scavenging of Antibiotic Resistant Bacteria. *Nanomaterials* 2021, *11*, 2829

**DOI:** 10.3390/nano13050824

**Published:** 2023-02-23

**Authors:** Lulu Liu, Wanfeng Wu, Yan Fang, Haoqiang Liu, Fei Chen, Minwei Zhang, Yanan Qin

**Affiliations:** 1 College of Life Science & Technology, Xinjiang University, Urumqi 830046, China; 2 Xinjiang Key Laboratory of Biological Resources and Genetic Engineering, Urumqi 830046, China

## Error in Figure

In the original publication [1], there was a mistake in Figure 6A II as published. A duplicate of the image for Figure 6A III was selected in error. The corrected Figure 6A II appears below. The scientific conclusions are unaffected. This correction was approved by the Academic Editor. The original publication has also been updated.

Original image (incorrect one):


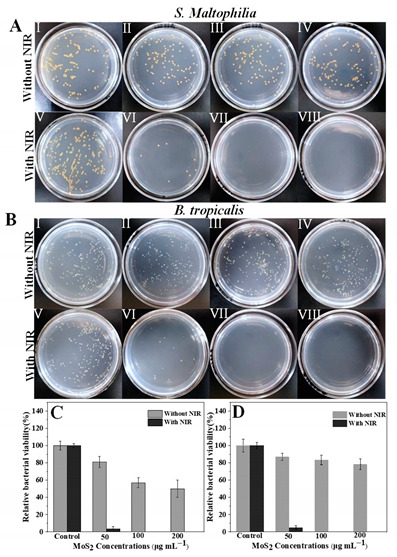


Corrected Figure 6:

**Figure 6:**
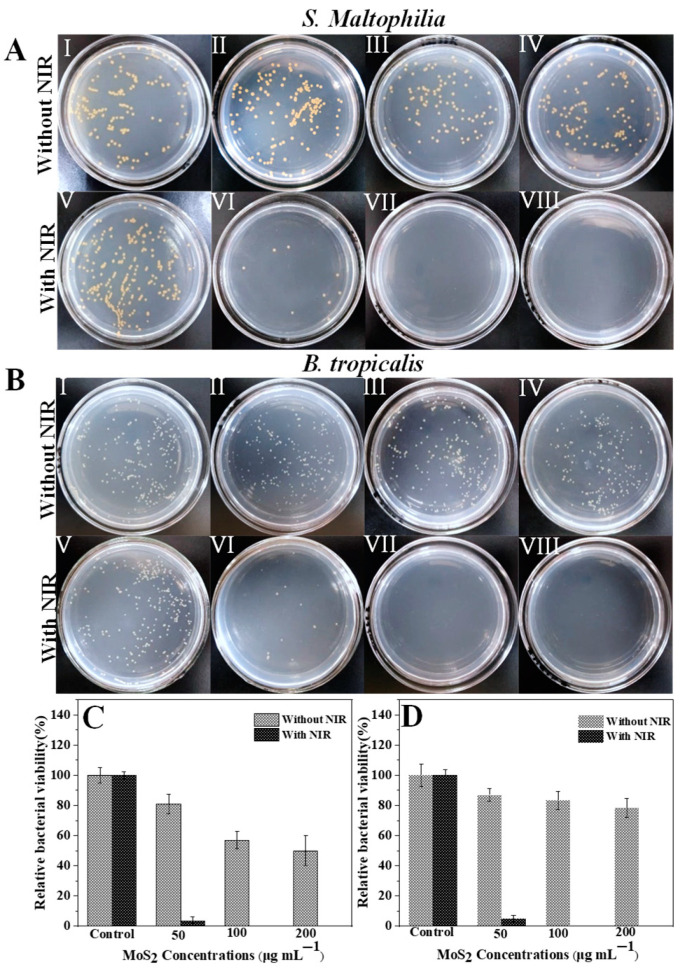
Photographs of bacterial colonies formed by (**A**) *S. Maltophilia* and (**B**) *B. tropicalis* after exposed to (I) PBS, (II) 50 μg/mL PEG-MoS_2_ NFs, (III) 100 μg/mL PEG-MoS_2_ NFs, (IV) 200 μg/mL PEG-MoS_2_ NFs, (V) PBS + NIR, (VI) 50 μg/mL PEG-MoS_2_ NFs + NIR, (VII) 100 μg/mL PEG-MoS_2_ NFs + NIR, (VIII) 200 μg/mL PEG-MoS_2_ NFs + NIR; The relative viability of (**C**) *S. Maltophilia* and (**D**) *B. tropicalis* after heat treatment with 808 nm NIR light for 10 min was measured by plate counting (Error bars are standard deviations of three parallel experiments).
