# Supplementary material for: Functionalized MoS2 Nanoflowers with Excellent Near-Infrared Photothermal Activities for Scavenging of Antibiotic Resistant Bacteria
Source: Nanomaterials (Basel). 2021 Oct 25;11(11):2829. doi: 10.3390/nano11112829 (PMC8622428; doi:10.3390/nano11112829)
Supplement: Supplementary file 1 [file nanomaterials-11-02829-s001.zip › nanomaterials-1427841-SI.pdf]

Supplementary Materials

Functionalized MoS_2 Nanoflowers with Excellent Near-Infrared Photothermal Activities for Scavenging of Antibiotic Resistant Bacteria

Lulu Liu [1,2,†], Wanfeng Wu [1,2,†], Yan Fang [1,2], Haoqiang Liu [1,2], Fei Chen [1,2], Minwei Zhang [1,2,*] and Yanan Qin [1,2,*]

[1] College of Life Science & Technology, Xinjiang University, Xinjiang 830046, China;
202112544@mail.sdu.edu.cn (L.L.); wwfeng1207@sina.com (W.W.);
fangyan1227@sina.com (Y.F.);
17699101675lhq@sina.com (H.L.); chen970701@sina.com (F.C.)
[2] Xinjiang Key laboratory of Biological Resources and Genetic Engineering, Xinjiang 830046, China
* Correspondence: zhangmw@xju.edu.cn (M.Z.); qin@xju.edu.cn (Y.Q.)
† Those authors equally contributed to this paper.

Methods

1. Apparatus

Scanning electron microscopy (SEM) images were obtained with the Hitachi S-4100 (Japan). The surface valence states of the samples were determined by X-ray photoelectron spectroscopy (XPS, ESCALAB250Xi). Absorption spectra were recorded on a UV-3100 PC spectrophotometer (Mapada Instrument Co., Shanghai, China). Near infrared exciter (MDL-H-808-5W- BE50906) was purchased from Changchun Optoelectronics Technology Co, Ltd. The size, shape and distribution of PEG-MoS_2 NFs were confirmed through transmission electron microscopy (TEM) images using a JEM-2100 instrument (JEOLCo., Japan) operated at an accelerating voltage of 200 kV. The ultrasonic treatment was performed using an SB-5200 DTD ultrasonicator (Scientz Biotechnology Co., Ningbo, China). The centrifugation was performed on a TDL-5 M refrigerated centrifuge (Shuke Instrument Co., Sichuan, China). The samples were dried out on a vacuum freeze-drying machine produced by Beijing Boyikang Experimental Instrument Co., Ltd.

2. Calculation of the photothermal conversion efficiency (η) of PEG-MoS_2 NFs

Photothermal properties of the PEG-MoS_2 NFs were measured according to the previous reports [1,2]. 1 mL of PEG-MoS_2 NFs dispersion with a concentration of 400 μg/mL was irradiated vertically at a power density of 1.5 W/cm^2 NIR (808 nm) for 16 min, and then the laser was shut off to bring the temperature down to room temperature. The digital thermometer records the temperature every 10 seconds. Then, the η value was calculated according to Eq. 1:

$$\eta=\frac{hS(T_{max}-T_{surr})-Q_{dis}}{I\ (1-10^{A}\lambda)} \quad (1)$$

Where h and S respectively represent the heat transfer coefficient and the surface area of the container, Tmax and Tsurr are the equilibrium temperature and the temperature of the surrounding environment, Qdis represents the heat-related to light absorption, I expresses the

incident laser power, and A_λ is the absorbance of PEG-MoS_2 NFs at 808 nm. The value of hS is calculated from Eq. 2:

$$hS=\frac{mC_p}{\tau_s} \qquad (2)$$

where m is the mass of the warming solution, Cp is the specific heat capacity of water ($\mathbf{4.2 \times 10^3}$J/(Kg·℃)), and to obtain the value of hS, a dimensionless temperature driving force θ is introduced to define $\boldsymbol{\tau_s}$ as follows. The value of θ is calculated from Eq. 3:

$$\theta=\frac{T-T_{surr}}{T_{max}-T_{surr}} \qquad (3)$$

In the cooling phase of the solution dispersion, the cooling time t and θ obey Equation 4, and the time constant ($\boldsymbol{\tau_s}$) in the heat transfer system can be determined ($\boldsymbol{\tau_s}$ is solved by the temperature change during cooling of the PEG-MoS_2 NFs dispersion(Fig.4B)) as follows.

$$t = -\tau_s \ln(\theta) \qquad (4)$$

Qdis in Equation 1 is the baseline energy. The value of Qdis is calculated from Eq. 5:

$$Q_{dis}=hS(T_{max,\ water}-T_{surr,water}) \qquad (5)$$

The time constant was τs = 435.36 s based on the linear fit from the cooling period. Accordingly, the photothermal conversion efficiency calculated by Eq. 5 and Eq.1 was η =30.6%.

3. Acid and gas production test

Bacterial cultures were incubated for 5 days at room temperature in Hugh Lifson's liquid medium containing bromothymol blue solution and Duchenne tubes, and observed daily for color change and bubble production in the tubes. If the color changed from blue to yellow, the bacteria were acid-producing bacteria, and if there were bubbles in the tube, the bacteria were gas-producing bacteria.

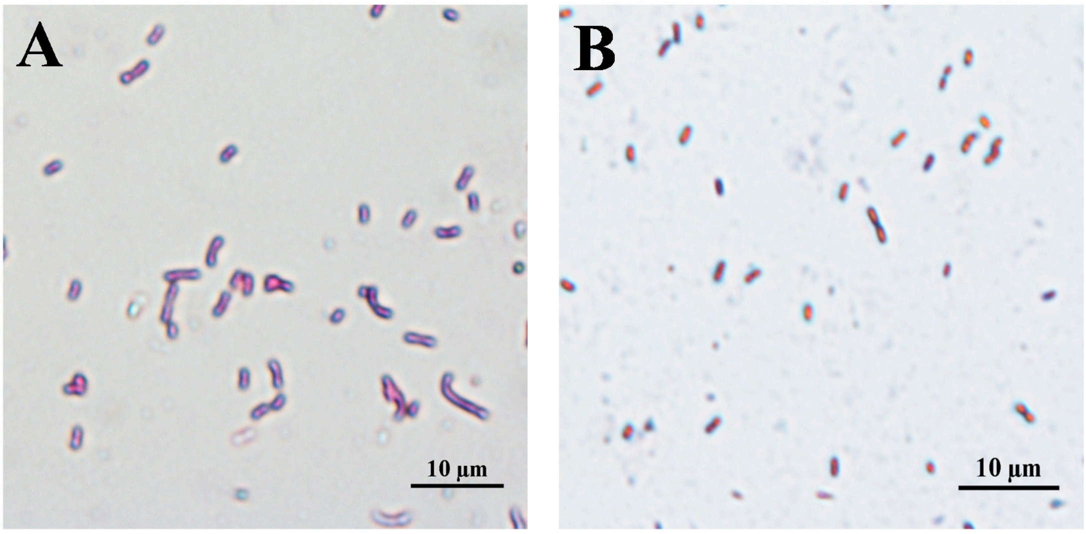

Figure S1. The gram staining results of Bacillus tropicus (**A**) and Stenotrophomonas maltophilia (**B**).

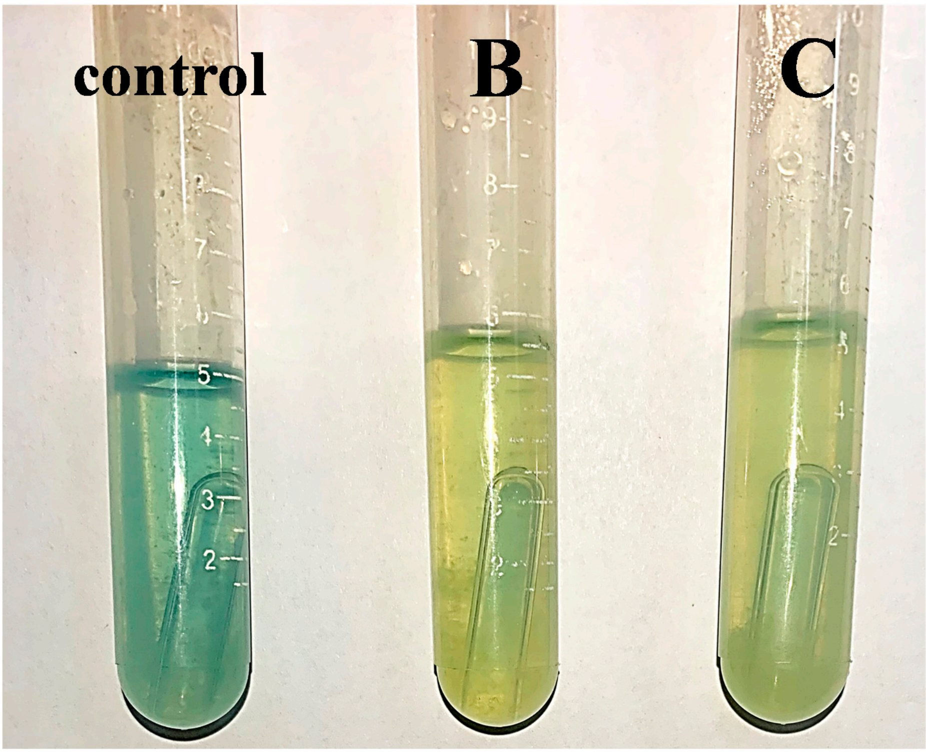

Figure S2. The acid and gas production test of Bacillus tropicus (B) and Stenotrophomonas maltophilia (C).

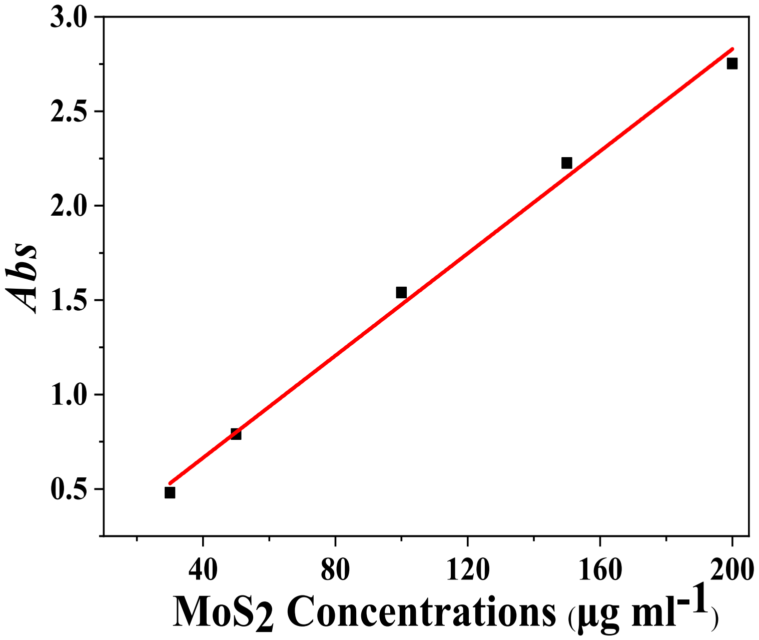

Figure S3. Linear relationship between the concentration of PEG-MoS_2 NFs dispersion and absorbance values at 808 nm.

References

1. Yu, J.; Yin, W.; Zheng, X.; Tian, G.; Zhang, X.; Bao, T.; Dong, X.; Wang, Z.; Gu, Z.; Ma, X.; Zhao, Y. Smart MoS_2/Fe3O4 nanotheranostic for magnetically targeted photothermal therapy guided by magnetic resonance/photoacoustic imaging. Theranostics. 2015, 5, 931-945, doi: 10.7150/thno.11802.
2. Zhang, X.; Zhao, Z.; Yang, P.; Liu, W.; Fan, J.; Zhang, B.; Yin, S. MoS_2@C nanosphere as near infrared / pH dual response platform for chemical photothermal combination treatment. Colloids Surf. B Biointerfaces. 2020, 192, 111054, doi: 10.1016/j.colsurfb.2020.111054
